# *‘If it don't fit, don't force it?’* If real‐world, complex clinical decisions are intrinsically categorical can dimensional systems add value? Reflections on Lahey, Tiemeier & Krueger (2022)

**DOI:** 10.1002/jcv2.12120

**Published:** 2022-11-26

**Authors:** Edmund J. S. Sonuga‐Barke

**Affiliations:** ^1^ Institute of Psychiatry, Psychology & Neuroscience King's College London London UK

Polemic is underappreciated as a form of scientific discourse, but it can serve a really important function. Unfettered by the need for balance, and often using language that is more emotive than would normally be seen in academic writing, authors can work in broad strokes to ruffle the feathers of the received wisdom of orthodox thinkers in ways that get them asking new questions. It can also help clarify the thinking of both those sympathetic to their cause and those opposed. In this sense the piece by Lahey et al. (2022; LTK), three of the most eminent researchers in our field, is great and glorious polemic. The mission of these scientific collosi—and there could be none more consequential—is nothing less than the complete overthrow of the categorical diagnostic system as set out in DSM and ICD. A system that up to this day dominates psychiatry and clinical psychology research and practice. For the authors much of their assault on the psychiatric status quo is based on rather dry and dusty statistical assertions; mental health and neuro‐developmental conditions are better characterised as dimensions rather than categories (reason 1); that, as a result, dimensional measures are more reliable (reason 2) and valid (reason 3); that dimensional systems deal better with the heterogeneity within and the overlap between different disorder classes (reason 4) or the way they change overtime (reason 6). However, their real passion, it seems to me, is reserved for when they talk of their antagonism towards the medical model. They argue that this is more than bad because it reifies difference as disorder or diseases and reinforces notions that young people suffering mental health and neuro‐developmental problems have a ‘sick brain or mind’. Crucially they see these medical models as perpetuating stigma and prejudice to which young people with mental health and neuro‐developmental conditions are often exposed. This they argue has the potential to worsen their suffering and reduce the chances of engagement with effective support, help and/or treatment. The abandonment of the medical model is the real ‘end’ here, the over‐turning of categorical diagnostic systems the ‘means’.

This is powerful and moving stuff. Of course, given its polemic purpose it's no surprise that it raises many vital questions that to my knowledge have not yet been fully resolved—in this way it does a great job in stimulating our thinking about what types of research needs to be undertaken before the change LTK want, can be implemented. Example questions include the following. Does diagnosis necessarily always increase stigma? Does a dimensional system handle heterogeneity and overlap better than a categorical one? Are there biological subtypes of conditions (starting to be identified, but yet to be formally classified) that would meet the statistical criteria for categories? Will replacing the concept of ‘mental disorder’ with the concept of ‘psychological problems’ lead to a trivialising or minimising of the impact of the chronic and serious conditions it is intended to characterise? Are the same dimensional concepts valuable for all psychological problems/mental disorders equally? What would the core problem/disorder dimensions be?

Important as providing answers to these and related questions is, in this commentary I will rather cut straight to what I believe is the question that captures the nub of the issue on which the *LTK* manifesto stands or falls. Can clinical decision making about complex psychological conditions ever be anything other than categorical in nature—driven as it is by practical conditionalities and psychological constraints? If it turns out that the answer to this question is that ‘no, such decisions are inevitably categorical’, even if the data from mental health research tell us that the underlying structures of the phenomena being characterised are in reality dimensional in nature, this would scupper the practical implementation of the LTK manifesto. More generally, and perhaps importantly, it would also challenge simplistic translational notions of the relationship between science and clinical practice.

Over the years I have written on numerous occasions about the relationship between science and clinical practice in our field from a philosophical perspective (Sonuga‐Barke, 1994; Sonuga‐Barke, 1998; Coghill & Sonuga‐Barke, 2010; Posner et al., 2020). Broadly speaking, my thesis has been that in clinical domains the structure and practice of science is rightly shaped by the clinical imperative which it serves: In our case to improve the lives of people living with, or at risk from, mental health and neurodevelopmental conditions. This ‘shaping’ works in both directions—constraining both the sort of science conducted *and* the way research findings are translated. With regard to the former, I have argued that the meta‐theory enshrined within the DSM/ICD diagnostic categories of mental disorder (and the assumption it embodies) derived from a history of many decades of clinical practice has determined our scientific paradigm (Sonuga‐Barke, 1998). In this way it has fundamentally shaped, some would say distorted, our research agenda in terms of the questions we have asked, the methods we have used and the way we have interpreted our data (Sonuga‐Barke & Castellanos, 2005). In a recent editorial I summarised my thinking on this matters by asking—*Are mental disorder diagnostics ripe for a Kuhnian revolution?* (Sonuga‐Barke, 2020). I questioned whether the assumptions that underpin the disorder paradigm are reconcilable with the scientific data emerging about the nature of these conditions—especially in terms of categories versus dimensions, psychological function versus dysfunction, and causal singularity versus causal heterogeneity; many points resonating strongly with the LTK manifesto.

However, while recognising the case for a shift in research paradigm, I highlighted some complications that might arise in bringing this change about that could risk undermining the vital link between science and clinical practice—cutting the former adrift from its translational purpose and the latter from it science‐driven basis. This I argued is because it risks breaking the bridge of communication between clinic and lab made possible by shared concepts and terms. This, I continued, would be the case if clinical decision making is inherently categorical in nature (i.e., contradicting the science which highlights its dimensionality). In addressing this possibility (before it can be ruled out as a constraint on progress to the dimensional model) three salient points should be considered. These relate to, (i) the practical nature of the clinician's task; (ii) the complex nature of the decisions being made and; (iii) the cognitive and psychological factors affecting the decision maker. With regard to point (i), workaday diagnosis is a very practical task—involving getting the best possible help and support to all those individuals that really need it, so improving the lives of people suffering from mental health and neuro‐developmental conditions. Whichever way you cut it this involves making a (or series of) binary distinction(s) between those that are judged to need and/or would benefit from a certain clinical intervention and those that would not—leaving aside all other resource‐related considerations. In this sense dimensional models inevitably collapse into binary judgements when practical clinical decisions have to be made. With regard to point (ii), such judgements are complex, carried out, as they are, within a multi‐dimensional decision space. Rarely can they be reduced to an individual's position on a single continuum of symptom severity. Rather, they involve the clinical interpretation of these symptoms in the light of a broader set of characteristics. Such decisions are aided by the detailed considerations described in diagnostic systems. In this way they are shaped by collective clinical and research knowledge built up over many years interpreted in the light of clinical training and experience. For instance, interpreting the clinical meaning of threshold‐level ADHD symptoms, and making sure they lead to the right therapeutic response, requires their contextualisation in terms of their course and persistence, their pervasiveness, their age appropriateness and, of course, their impact on functioning. It is also important to rule out that they are not being confused with another condition (Posner et al., 2020). In this sense clinical decision making is complex decision making, and when making complex decisions humans fall back on the use of heuristics or rules of thumb especially in highly resource poor settings typical in clinical practice (constrained finance, time, decision processing limitations). It's likely that such heuristics will take on a categorical quality as clinicians pattern match their patient's characteristics to the diagnostic archytype (Sonuga‐Barke, 1998). With regard to the final‐point I also argued that current diagnostic approaches cut with the psychological grain of human social perception especially with regard to our tendency to impose categorical structures onto dimensional stimuli and to relate these to individual differences as traits (Sonuga‐Barke, 1998).

Clearly scientific study leading to the practical resolution of these potential constraints on the nature of clinical decision making will be essential before the sort of dimensional system proposed in the LTK can be designed and implemented. If it turns out the decision making is intrinsically categorical, and despite our best efforts we have to stick with systems very like the current one for clinical purposes—then we are faced with the really knotty conundrum: That, because of their different purposes, science and clinical practice require different ways to think and talk about the same phenomenon. If this were to be the case, it would obviously complicate the translational mission of science vis‐à‐vis clinical practice by weakening that conceptual bridge of meaning that shared clinical concepts and terms bring which currently allow clinical questions to be interpretable scientifically and scientific data to be implementable clinically. Clearly a system that maintains the scientific integrity of the field while promoting the practical application of knowledge will be required (Sonuga‐Barke et al., 2022).

## AUTHOR CONTRIBUTIONS

**Edmund Sonuga‐Barke**: Conceptualization; Writing—original draft; Writing—review & editing.

## CONFLICT OF INTEREST

The author has declared that he has no competing or potential conflicts of interest.
